# Designing Analogs of SAAP-148 with Enhanced Antimicrobial and Anti-LPS Activities

**DOI:** 10.3390/ijms252111776

**Published:** 2024-11-01

**Authors:** Lingmin Gan, Yulang Chi, Yunhui Peng, Subo Li, Hongwei Gao, Xue Zhang, Shouping Ji, Zili Feng, Shikun Zhang

**Affiliations:** 1 Academy of Military Medical Science, Beijing 100850, China; glm1632024@163.com (L.G.); 18759705100@163.com (Y.P.); lisubo2007@sina.com (S.L.); gaohongwei1976@126.com (H.G.); zhangxue19900121@126.com (X.Z.); 2 School of Biological Science and Engineering, Shaanxi University of Technology, Hanzhong 723001, China; 3 College of Oceanology and Food Science, Quanzhou Normal University, Quanzhou 362000, China; ylchi@qztc.edu.cn (Y.C.); 20801@qztc.edu.cn (S.J.)

**Keywords:** antimicrobial peptides, SAAP-148, Lys-scan, cytotoxicity, antimicrobial activity

## Abstract

SAAP-148, a derivative of LL-37, exhibits a well-defined amphipathic structure and enhanced antimicrobial activity; however, it also displays significant cytotoxicity towards human cells. In this study, we employed Lys-scan to produce a series of amphiphilic SAAP-148 analogs derived from the SAAP-148 sequence to investigate the impact of the distribution of positively charged residues on the biological viability of the antimicrobial peptides (AMPs). The physical properties and biological activity of the designed peptides were subsequently compared. The substitution of lysine resulted in an increase in the overall charge of SAAP-148 and a decrease in its overall hydrophobicity and hyd. moment, except for SAAP-10 where an analogue substitution occurred at the 18th residue. The replacement of lysine led to a reduction in hemolytic activity compared to SAAP-148, with slightly higher haemolysis rates observed in SAAP-11 and SAAP-13. The cytotoxicity of peptides towards human normal lung epithelial cells (BEAS-2B) was closely linked to their haemolytic activity, indicating that substituting lysine may mitigate the cytotoxic effects of SAAP-148. Additionally, the arrangement of positively charged residues in the peptides significantly influenced its antimicrobial activity. Our findings suggest that the positioning of a positively charged residue has a significant impact on the biophysical properties of the peptide. Additionally, the substitution of lysine at different positions demonstrates an influence on the anti-lipopolysaccharide (anti-LPS) activity of SAAP-148. These discoveries provide valuable insights for the design and optimization of antimicrobial peptides, which will be advantageous for the future development of antimicrobial agents.

## 1. Introduction

Antibiotics represent one of the most efficacious modalities of chemotherapy and are widely regarded as a paramount medical advancement of the twentieth century, potentially even within the annals of medical history. However, their efficacy is threatened by the uncontrolled spread of antibiotic resistance throughout microbial populations. The most prominent of these antibiotic resistance bacteria are the ESKAPE pathogens, which includes *Enterococcus faecium*, *Staphylococcus aureus*, *Klebsiella pneumoniae*, *Acinetobacter baumannii*, *Pseudomonas aeruginosa*, and *Enterobacter species* [1]. According to the report from the Centers for Disease Control and Prevention (CDC), more than 2.8 million antibiotic-resistant infections occur in the United States each year, resulting in over 35,000 deaths [2]. The World Health Organization (WHO) has forecasted that if the current situation persists, antibiotic resistance will emerge as the leading cause of mortality by 2050, resulting in an estimated annual toll of 10 million deaths [3]. Hence, it is crucial to discover novel antibacterial agents with a distinct mechanism of action to address the challenge of antibiotic-resistant bacterial infections.

AMPs, also referred to as host defense peptides, are widely distributed among animal, insect, and plant species and play a pivotal role in their innate immune systems [4,5]. AMPs typically consist of short polypeptides comprising 10–50 amino acids and may possess a cationic, anionic, or neutral charge [6,7]. While the precise mechanism of action for some AMPs remains to be fully elucidated, the majority of AMPs exhibit amphipathic and cationic properties [7], enabling them to interact with the negatively charged outer surface of bacteria and subsequently induce depolarization, permeabilization, and disruption of the cytoplasmic membrane [8]. The mechanism of action of AMPs provides a distinct advantage over traditional antibiotics by effectively inhibiting the emergence of drug resistance. Consequently, AMPs have been recognized as promising candidates for the development of innovative drugs targeting antibiotic-resistant pathogens [9,10]. AMPs offer a multitude of advantages over antibiotics, encompassing a broad spectrum of antimicrobial activities that include antifungal, antiviral, and antibacterial properties [11]. Nevertheless, their clinical application continues to pose challenges such as potential human toxicity, susceptibility to degradation under harsh environmental conditions (due to sensitivity to proteases and extreme pH), and lack of specificity for particular strains. These limitations constrain their utility in drug development despite the absence of reported contributions to resistance development [12].

AMPs exhibit a diverse array of primary and secondary structures, and comprehending their structural characteristics is imperative for their advancement as therapeutic agents [13,14]. In this study, we aimed to explore the potential of SAAP-148 and its analogs for various applications, particularly focusing on their suitability for topical use. Numerous investigations have been undertaken to augment the efficacy of natural AMPs against specific pathogens while minimizing toxicity at therapeutic levels [12,15]. These peptides possess amphiphilic properties, solubility in aqueous environments, and the ability to partition into lipid bilayers due to the spatial segregation of hydrophilic and hydrophobic amino acids [16]. The degree to which water-soluble AMPs distribute into the lipid bilayer is dictated by their hydrophobicity, a crucial factor for membrane permeabilization. Excessive hydrophobicity can result in toxicity towards mammalian cells [13]. The structure–activity relationship of AMPs illustrates that properties such as charge, secondary structure, hydrophobicity, amphiphilicity, and hydrophobic torque are crucial for the specificity and biological activity of peptides. These properties are interconnected, so modifying one typically leads to significant changes in one or more other properties [17]. The presence of amphiphilicity allows for flexible conformations, leading to the formation of α-helices, β-sheets, or a combination of both when interacting with target microbial membranes. However, perfect amphipathicity has often resulted in a simultaneous increase in antimicrobial activity and cytotoxicity [18].

Several recent studies have suggested that the substitution of positively charged residues for the nonpolar face, in order to disrupt the α-helical amphipathic structure, is linked to a reduction in haemolytic activity while maintaining antimicrobial efficacy comparable to that of the unaltered peptide [16,19]. LL-37, derived from the human cationic antibacterial protein 18 kDa (hCAP18), plays a crucial role in the defense against both local and systemic infections. [20,21]. SAAP-148, a peptide derived from LL-37 with a well-defined amphipathic structure and enhanced antimicrobial activity, also demonstrates significant cytotoxicity towards human cells [22,23]. In this study, we employed a Lys-scan to generate a series of amphiphilic SAAP-148 analogs derived from the SAAP-148 sequence. The physical properties of the designed peptides were computed using HeliQuest [24]. Subsequently, we assessed the antimicrobial activity against three Gram-positive and two Gram-negative bacteria, as well as the anti-LPS activity of the peptides. Additionally, we determined the haemolytic properties and cytotoxicity to BEAS-2B cells. The findings indicated that the positioning of a positively charged residue has a significant impact on the biophysical properties of SAAP-148.

## 2. Results

### 2.1. Designing and Forecasting the Physicochemical Properties of Peptides

The side chains of amino acids play a pivotal role in determining their physical and chemical properties. While alanine scanning is commonly utilized for investigating the impact of different amino acids on antimicrobial peptide activity, our study employed lysine scanning, a polar hydrophilic amino acid, to evaluate the influence of antibacterial activity and cytotoxicity against eukaryotic cells with the disruption of amphiphilicity at various positions of SAAP-148. The sequences and molecular weights of these analogues are outlined in Table 1. The strong agreement between the measured and theoretical molecular weights of the peptides indicates precise synthesis of the compounds (Figures S3 and S4).

**Figure 1:**
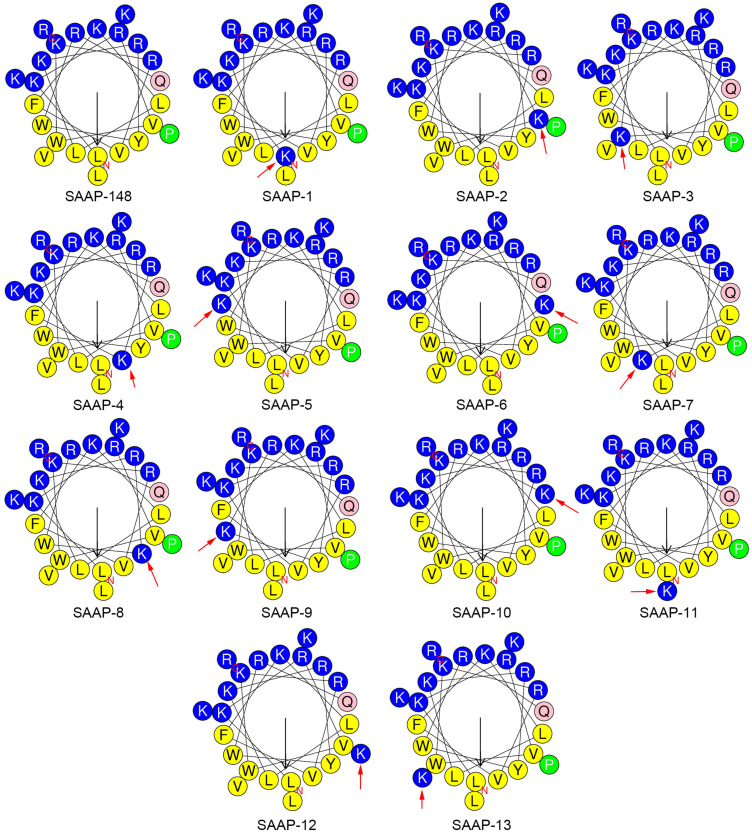
Helical wheel projections of SAAP-148 and its analogues generated using HeliQuest [24]. By default, the output presents the polar, positively charged amino acids as blue circles; the polar, uncharged amino acids as pink circles; the hydrophobic residues as green; and the most hydrophobic residues as yellow circles. The red arrow indicates the mutated residue.

The physicochemical parameters crucial for the antimicrobial activity of AMPs have been identified, encompassing net charge, helicity, hydrophobicity, and amphipathicity. The hydropathicity, mean relative hydrophobic moment, and charge of peptides were predicted using the HeliQuest (https://heliquest.ipmc.cnrs.fr/ (accessed on 12 March 2024)) [24]. Lysine is a hydrophilic amino acid, and its substitution leads to a decrease in the hydrophobicity of the SAAP-148 analogue. The anticipated results are consistent, with the degree of reduction in hydrophilicity varying depending on the position at which lysine is substituted (Table 2). The amphipathicity of the peptides, as denoted by the μH (mean relative hydrophobic moment), is elaborated on in Table 2. The hydrophobic moment of SAAP-148 analogues was decreased except for SAAP-10, which is on account of the substitution occurring on the18th residue. (Figure 1) The 18th residue of SAAP-148 is glutamine, a polar amino acid residue, located at the junction of the hydrophobic and hydrophilic surfaces of the SAAP-148 α-helix. When this residue was replaced by lysine, the side chain of this lysine would aggregate with the hydrophilic surface of SAAP-148, hence the increase of the hydrophobic moment. The retention time data determined by reversed-phase high-performance liquid chromatography (RP-HPLC) reflects the hydrophobicity difference between peptide analogues. It is well documented that the formation of a hydrophobic-binding domain due to peptide secondary structure can affect peptide interactions with reversed-phase matrices. This effect was observed particularly for amphipathic α-helical peptides [25]. Given this preferred binding domain, amphipathic α-helical peptides are considerably more retentive than non-amphipathic peptides with the same amino acid composition.

### 2.2. Antimicrobial Activity of SAAP-148 and Its Analogues

The peptides were evaluated for their antimicrobial effectiveness against a panel of bacterial strains, as demonstrated by the determination of minimum inhibitory concentrations (MIC). SAAP-148 exhibited significant antimicrobial activity at concentrations ranging from 3.13 to 50 μM, surpassing that of LL-37 (refer to Table 3). *E. coli*, *P. aeruginosa*, and *S. epidermidis* demonstrated higher susceptibility to SAAP-148 and its analogues in comparison to *K. pneumoniae* and *S. aureus.* All analogues demonstrated comparable antimicrobial efficacy to SAAP-148, although SAAP-4, SAAP-7, SAAP-11, and SAAP-12 displayed diminished antimicrobial activity against *K. pneumoniae*. The geometric mean of minimum inhibitory concentration (MIC) values (GM) was calculated for both Gram-negative and Gram-positive bacteria, revealing that SAAP-6 exhibited the highest antimicrobial activity against both types of bacteria.

### 2.3. Haemolytic Activity of SAAP-148 and Its Analogues

**Figure 2:**
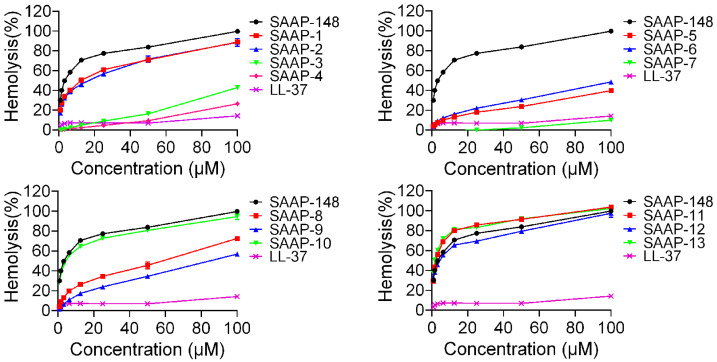
The hemolytic activity of SAAP-148 and its derivatives on human erythrocytes.

The haemolytic activities of the peptides against human erythrocytes were evaluated to ascertain their cytotoxicity towards mammalian cells. A summary of these activities is depicted in Figure 2. SAAP-148 demonstrated significantly higher hemolytic activity in comparison to LL-37, a well-studied human cathelicidin family member of AMPs. All analogues displayed reduced hemolytic activity relative to SAAP-148, with the exception of SAAP-11 and SAAP-13. SAAP-11 and SAAP-13 demonstrated slightly higher haemolysis rates compared to SAAP-148 (80.18% and 81.35% versus 70.62%, respectively, at concentrations of 12.5 μM). Conversely, SAAP-3–SAAP-9 exhibited significantly lower haemolysis rates than SAAP-148, with SAAP-7 displaying the lowest haemolytic activity at only 10.11% at a concentration of 100 μM. SAAP-1, SAAP-2, SAAP-10, and SAAP-12 exhibit comparable hemolytic activity to SAAP-148. The minimum haemolytic concentration (MHC) of peptides refers to the lowest concentration at which 10% haemolysis of human erythrocytes occurs, and it was introduced as a quantitative measure of peptide haemolytic activities. As shown in Table 4, seven analogues of SAAP-148 demonstrated higher MHC values compared to SAAP-148, with SAAP-4 and SAAP-7 exhibiting the highest value at 100 μM, representing a 133.33-fold increase over that of SAAP-148.

### 2.4. Cytotoxicity Activity of SAAP-148 and Its Analogues

**Figure 3:**
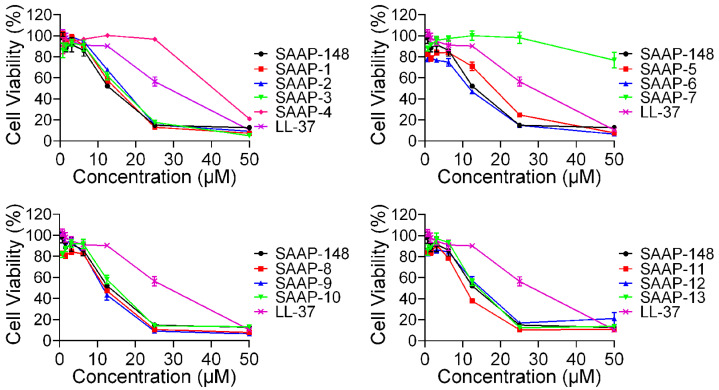
The cytotoxic effects of SAAP-148 and its analogues on BEAS-2B cells.

To further investigate the cytotoxicity of these peptides on mammalian cells, the CCK-8 assay was conducted on BEAS-2B cells, and the result was presented in Figure 3. SAAP-148 exhibited significantly greater cytotoxicity compared to LL-37, while all analogues demonstrated similar levels of cytotoxicity relative to SAAP-148, with the exception of SAAP-4 and SAAP-7. Specifically, cell viability following treatment with 25 μM concentrations of SAAP-4, SAAP-7, and SAAP-148 was 96.88%, 98.22%, and 14.9%, respectively. Similar to the findings of the haemolytic assay, it was observed that SAAP-7 demonstrated the least cytotoxicity towards BEAS-2B cells, with a recorded cell viability of 76.81% at a concentration of 50 μM.

### 2.5. The Therapeutic Index (TI) of SAAP-148 and Its Analogues

The geometric mean of minimum inhibitory concentration (MIC) values (GM) from the five strains was calculated to provide a comprehensive assessment of antimicrobial activity, as presented in Table 4. Six analogues demonstrated enhanced antimicrobial activity compared to SAAP-148, with SAAP-6 exhibiting the highest antimicrobial activity (3.59), representing a two-fold decrease in comparison to that of SAAP-148 (7.18). The therapeutic index (TI) is determined by the ratio of the minimum haemolytic concentration (MHC) of peptides to the geometric mean (GM) of peptides, and it serves as a parameter for evaluating the cell-selective toxicity of AMPs towards bacterial cells in comparison to mammalian cells. SAAP-148 exhibited a lower therapeutic index (TI) compared to LL-37. Among the ten analogues, there was an enhancement in TI relative to SAAP-148, while four analogues demonstrated a decrease in TI compared to SAAP-148. Notably, significant improvements in TI against the five bacterial strains were observed for SAAP-3, SAAP-4, and SAAP-7, with values of 72.73, 83.55, and 55.09, respectively.

### 2.6. Membrane Damage Induced by SAAP-148 and Its Analogues

Propidium iodide (PI) was utilized as a marker for assessing the structural integrity of cell membranes and the occurrence of cell death. The impact of peptides on BEAS-2B cells, *E. coli*, and *S. aureus* was investigated by treating the cells with peptides followed by PI staining and flow cytometry (FACS) analysis to evaluate peptide-induced membrane damage. Table 5 presents the results of PI staining in BEAS-2B cells (1 × 10^6^) following treatment with peptides at a concentration of 6.25 μM. The observed membrane damage induced by peptides in BEAS-2B cells exhibited a similar trend to the hemolytic activity and toxicity of the peptides. FACS analysis at a peptide concentration of 6.25 μM was employed to determine the membrane damage caused by peptides in *E. coli* and *S. aureus* cells (Table 5). SAAP-148 and its analogues were capable of inducing damage to both bacterial and eukaryotic cell membranes within 30 min, with SAAP-148 demonstrating the highest potential. The percentage of PI-positive cells in the SAAP-7-treated group was found to be 34.9% for *E. coli* and 21.1% for *S. aureus*, respectively.

### 2.7. Anti-LPS Property of SAAP-148 and Its Analogues

**Figure 4:**
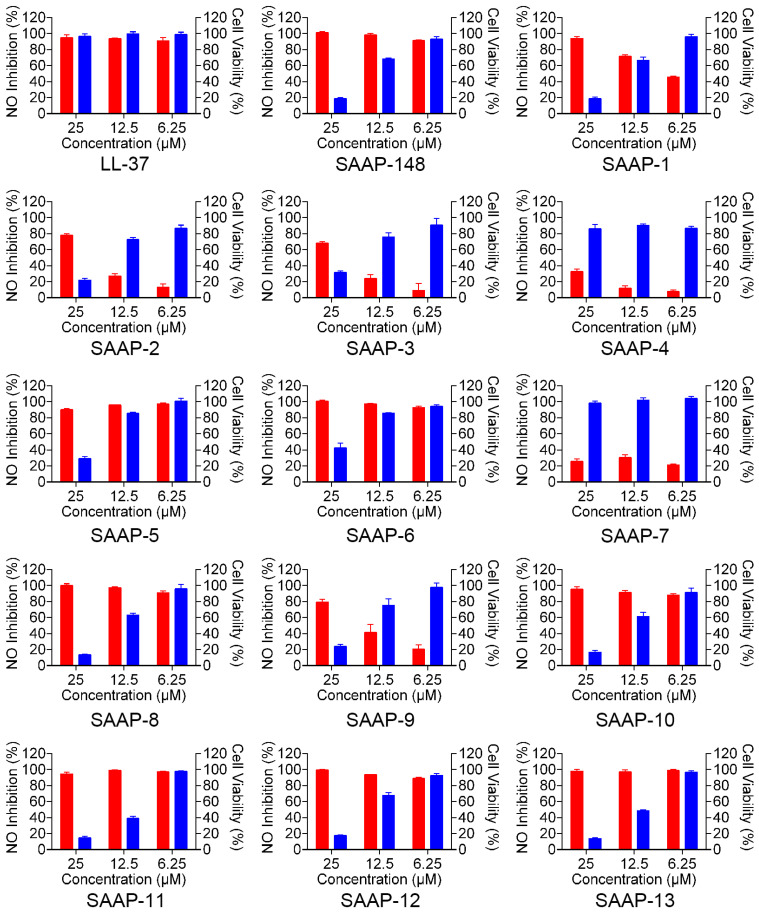
The effect of SAAP-148 and its analogues on the inhibition of LPS-induced NO in mouse RAW264.7 macrophages. The red-shaded column on the left-hand side of the figure presents the NO inhibition rate of SAAP-148 and its analogues. The blue-shaded column on the right-hand side of the figure presents the toxicity of SAAP-148 and its analogues.

LL-37 is a promising peptide for the regulation of inflammation and mitigation of endotoxin impact due to its ability to directly bind to LPS and counteract its biological effects, such as the inhibition of interleukin-6 (IL-6), interleukin 1β (IL-1β), and LPS-induced nitric oxide (NO). We conducted a series of experiments to assess the effectiveness of SAAP-148 and its analogues in reducing NO production by mouse mononuclear pacrophages cells (RAW264.7) after LPS stimulation. Initially, the toxicity of SAAP-148 and its analogues on RAW264.7 cells was assessed, revealing a dose-dependent toxicity within the concentration range of 6.25 to 25 μM for most analogues, with the exception of SAAP-4 and SAAP-7 (Figure 4, the blue-shaded column on the right-hand side of the figure). SAAP-148 and its analogues displayed negligible toxicity to RAW264.7 cells at a concentration of 6.25 μM. Subsequently, we employed a Griess assay to evaluate the levels of nitrite in RAW264.7 cells following treatment with SAAP-148 and its analogues, both with and without LPS treatment. Our findings indicate that SAAP-148 exhibits comparable efficacy to LL-37 in inhibiting NO production, while seven analogues demonstrated similar NO inhibition ability to that of SAAP-148 at 6.25 μM. However, six analogues displayed reduced NO inhibition ability compared to that of SAAP-148 at 6.25 μM (Figure 4, the red-shaded column on the left-hand side of the figure).

## 3. Discussion

The emergence of antibiotic-resistant clinical bacterial strains has prompted a shift in research focus towards innovative classes of antimicrobial compounds. AMPs represent a promising new category of antibiotics, thanks to their unique antimicrobial mechanism compared to traditional antibiotics [12]. While the precise mechanism of action of AMPs remains elusive, there is a suggestion that AMPs primarily target the cytoplasmic membrane. Consequently, it is improbable for microorganisms to develop resistance to membrane-active peptides, as this would necessitate significant alterations in the lipid composition of their cell membranes [26,27]. The therapeutic application of these antimicrobial peptides (AMPs) has been consistently impeded by a range of challenges, particularly with respect to their potential toxicity towards host cells [28,29]. Recent investigations have revealed that AMPs display conserved physical and chemical attributes; however, their antibacterial efficacy cannot be ascribed to a singular property but instead hinges on the precise amalgamation of multiple properties [30]. Numerous structure–function studies have been conducted on both natural and synthetic antimicrobial peptides to design AMPs that effectively target pathogens while minimizing toxicity at therapeutic doses. However, there is a lack of clear guidelines regarding the optimal number of hydrophobic or charged residues to maximize antimicrobial activity and minimize cytotoxicity in AMPs, as this varies significantly among different peptides.

Gram-negative bacteria are characterized by the presence of a plasma membrane and an outer membrane comprised of lipopolysaccharides with negative charges. In contrast, Gram-positive bacteria do not possess an outer membrane but instead have a cell wall layer consisting of peptidoglycan and negatively charged teichoic acid located outside the plasma membrane [31]. Hence, cationic AMPs are capable of engaging with the membrane via electrostatic attraction, establishing a robust foundation for subsequent membrane disruption and cytoplasmic entry. Peptides accumulate on the membrane surface following the initial interaction, undergo diffusion, and self-assemble upon reaching a certain concentration [16]. Upon surpassing a threshold concentration, the aggregated peptides induce membrane permeation, subsequently leading to membrane rupture [32].

This study utilized Lys-scan to generate a series of amphiphilic SAAP-148 analogs in order to investigate the impact of the distribution of positively charged residues on the biophysical properties and biological activities of the peptide. All SAAP-148 analogs contain an additional positively charged residue compared to SAAP-148, leading to enhanced peptide interaction with bacterial membranes. The hydrophobic nature of peptides significantly impacts their ability to cross the lipid bilayer [33]. However, an excessive level of hydrophobicity can lead to reduced antibacterial effectiveness and increased mammalian toxicity. Owing to their limited solubility in aqueous solutions, highly hydrophobic peptides are more prone to binding with and disrupting eukaryotic cell membranes [32,33]. SAAP-148 demonstrates the highest level of hydrophobicity among all tested peptides, with a calculated value of 0.30083. The decrease in peptide hydrophobicity can be attributed to the substitution of lysine, an amino acid with hydrophilic properties, resulting in an overall reduction in the hydrophobicity of SAAP-148. The extent of this reduction in hydrophilicity varies depending on the specific position at which lysine is replaced.

The amphiphilicity of the peptides is crucial for their ability to penetrate microbial membranes. Detailed information on the amphipathicity of the peptides, as indicated by μH (mean relative hydrophobic moment), can be found in Table 2. The substitution of lysine on the hydrophobic face of SAAP-148 led to a reduction in the peptide’s amphipathicity. The negatively charged residue Gln18, located at the interface of the hydrophobic and hydrophilic regions of the SAAP-148 α-helix, plays a critical role in modulating the hydrophobic moment. Substituting this residue with lysine leads to aggregation of the lysine side chain with the hydrophilic surface of SAAP-148, resulting in an overall increase in the hydrophobic moment. The presence of perfect amphipathicity has typically led to a simultaneous increase in antimicrobial activity and cytotoxicity18. SAAP-10 may exhibit potent antimicrobial and hemolytic properties. However, the current investigation reveals that SAAP-10 with enhanced amphipathicity demonstrates reduced hemolytic activity. The results indicated that amphipathicity does not emerge as the most critical parameter for these peptides.

It is widely recognized that the emergence of a hydrophobic-binding domain due to peptide secondary structure can significantly influence the interactions of peptides with reversed phase matrices (the peptide retention time). This phenomenon was particularly evident in the case of amphipathic α-helical peptides [34]. Owing to their preferred binding domain, amphipathic α-helical peptides demonstrate significantly greater retention compared to non-amphipathic peptides with identical amino acid composition. This discrepancy may be attributed to the lack of a well-defined α-helical structure in SAAP-148 and its analogues (Figure S1) [35].

Increasing the abundance of positively charged residues is advantageous for the initial electrostatic interactions between AMPs and negatively charged components of bacterial membranes, thereby enhancing selectivity [16]. Nevertheless, our findings suggest that certain analogues with elevated levels of positively charged residues demonstrated heightened antimicrobial efficacy against the bacteria (Table 3). SAAP-148-6 demonstrates the most potent antimicrobial activity with the lowest GM value among all analogues. However, certain analogues exhibited insensitivity to specific bacteria. For example, the MIC of SAAP-4 and SAAP-7 against *K. pneumoniae* was 100 μM, in contrast to 6.25 μM for SAAP-148. As a result, the GM value of these two peptides was higher than that of SAAP-148. In addition, the membrane damage caused by peptides to *E. coli* and *S. aureus* cells was consistent with the results of the MICs. SAAP-7 exhibited a lower capacity for membrane damage compared to its analogues, in line with its antimicrobial activity. The MIC of SAAP-7 against *E. coli* and *S. aureus* was 100 μM, indicating a low potent antimicrobial effect on both bacterial strains. These findings lead to the conclusion that the arrangement of positively charged residues in the peptide significantly influences its antimicrobial activity.

The haemolytic activity of the peptides against human erythrocytes was employed as a primary indicator of peptide toxicity towards higher eukaryotic cells, rendering it an attractive avenue for antimicrobial peptide research and development in order to achieve optimal antimicrobial efficacy with minimal host toxicity [36]. Our findings suggest that the replacement of lysine led to a decrease in hemolytic activity compared to SAAP-148, with the exception of SAAP-11 and SAAP-13, which exhibited slightly higher haemolysis rates. Conversely, SAAP-3 to SAAP-9 demonstrated significantly lower haemolysis rates than SAAP-148, with SAAP-7 displaying the lowest hemolytic activity at only 10.11% at a concentration of 100 μM. The MHC was introduced as a quantitative measure of peptide haemolytic activities. Table 4 demonstrates that six analogues showed MHC values similar to SAAP-148, while seven analogues of SAAP-148 exhibited higher MHC values. These results suggest that the substitution of lysine may attenuate the haemolytic activity of SAAP-148. The cytotoxicity of peptides towards BEAS-2B cells was found to be closely correlated with their haemolytic activity outcomes. The damage to BEAS-2B cell membranes caused by the peptides displayed a similar pattern to the peptides’ haemolytic activity and toxicity, with SAAP-7 exhibiting the lowest potential.

The therapeutic index (TI) is a widely utilized parameter for indicating the specificity of antimicrobial agents, which is determined by the ratio of MHC (hemolytic activity) and MIC (antimicrobial activity). Consequently, larger TI values signify greater antimicrobial specificity [37,38]. The TI can be enhanced through one of three mechanisms: increasing antimicrobial activity, reducing hemolytic activity while maintaining antimicrobial activity, or a combination of both augmenting antimicrobial activity and diminishing hemolytic activity. SAAP-148, a peptide with potent antimicrobial activity and significant haemolytic activity, exhibited a lower therapeutic index (TI) compared to LL-37 (0.11 and 3.48 for SAAP-148 and LL-37, respectively). Seven analogues demonstrated an improvement in therapeutic index (TI) compared to SAAP-148, while four analogues showed a decrease in TI relative to SAAP-148. Notably, substantial enhancements in TI against the five bacterial strains were observed for SAAP-3, SAAP-4, and SAAP-7, and the increased therapeutic index was primarily attributed to the reduction of haemolytic activity. Specifically, enhanced antimicrobial activity and reduced haemolytic activity were observed for SAAP-3. The lysine substitution at Gln18, Leu19, Pro22, and Val23 of SAAP-148 led to a reduction in antimicrobial activity while maintaining comparable haemolytic activity to that of SAAP-148. These analogues exhibited a decreased therapeutic index (TI) compared to SAAP-148. The findings suggest that the location of a positively charged residue has an impact on the biophysical characteristics and selectivity of the peptide.

LPS acts as a barrier to prevent peptides from integrating into the inner membrane and is considered the primary target for antimicrobial peptides [39,40]. In addition to its direct antimicrobial activity, many AMPs often demonstrate immunomodulatory activities, such as inducing chemokines and neutralizing endotoxins to inhibit LPS-induced pro-inflammatory cytokine production. Cationic antimicrobial peptides (AMPs) engage in strong electrostatic interactions with negatively charged LPS [41,42]. LL-37 demonstrates remarkable efficacy in reducing LPS-induced nitric oxide (NO) production while displaying minimal cytotoxicity. SAAP-148 has exhibited potential in decreasing NO production induced by LPS, but it also shows toxicity against RAW264.7 cells. The NO inhibition activity of SAAP-148 and its analogues varies, despite possessing the same positively charged residues. These findings suggest that lysine substitution at different positions in SAAP-148 impacts its anti-LPS capability.

## 4. Materials and Methods

### 4.1. Materials and Regents

The LL-37, SAAP-148 and its analogues peptides were synthesized according to the standard Fmoc procedure. The Fmoc-protected resin and monomers are first treated with a basic solvent (piperidine) to remove the amino protecting groups. The carboxyl group of the next amino acid is activated by an activator. The activated monomer reacts with the free amino group to form a peptide bond. These two steps are repeated in a cycle until the synthesis is complete. After synthesis, the peptide is eluted from the column and purified by reverse-phase semipreparative high-performance liquid chromatography as detailed in Table 1. The peptides were dissolved in PBS to create a 1000 μmol/L stock solution for subsequent use. The purity of the synthetic peptide exceeded 95%. Blood samples from healthy donors were obtained from the Fifth Medical Center of Chinese PLA General Hospital (Beijing, China). Human normal lung epithelial cells (BEAS-2B) and mouse mononuclear macrophages cells (RAW 264.7) were sourced from Beyotime Biotechnology (Beijing, China), along with the Enhanced Cell Counting Kit-8 (CCK-8) and nitric oxide detection kit. RPMI-1640 and DMEM were procured from Merck (Darmstadt, Germany), while FBS was obtained from ExCell Bio (Shanghai, China). LPS-B5 (from *E. coli* 055:B5) was procured from Invivogen (Toulouse, France). Five bacterial strains, including *Escherichia coli* (1.8732), *Pseudomonas aeruginosa* (1.2421), *Klebsiella pneumonia* (1.1736) as Gram-negative bacteria, and *Staphylococcus aureus* (1.8721) and *Staphylococcus epidermidis* (1.4260) as Gram-positive bacteria, were procured from the China General Microbiological Culture Collection Centre (CGMCC) (Beijing, China).

### 4.2. Circular Dichroism (CD) Spectra and Physicochemical Properties Forecasting of the Peptides

CD spectra (Figure S1) were acquired using a JASCO J-1500 spectropolarimeter (JASCO Inc., Easton, MD, USA) in 0.1 cm pathlength cells under nitrogen at 25 °C. The spectra were recorded in the range of 190 to 250 nm at a peptide concentration of 100 μM in SDS. Secondary structure was determined using the BeStSel server [43]. Hydropathicity, mean relative hydrophobic moment, and charge of peptides were predicted using the HeliQuest (https://heliquest.ipmc.cnrs.fr/ (accessed on 12 March 2024)) [24]. The 3D structure of SAAP-148 and its analogues (Figure S2) was predicted using PEP-FOLD4 servers (https://bioserv.rpbs.univ-paris-diderot.fr/services/PEP-FOLD4/ (accessed on 19 June 2024)) [35].

### 4.3. Haemolytic Activity of the Peptides

The haemolytic activity of the peptides was evaluated using a standard procedure with minor adjustments [19]. Fresh human erythrocytes were washed thrice and subsequently resuspended at 1.25% haematocrit in PBS. Forty microlitres PBS-diluted peptide solution was added to a 96-well plate (Corning Inc., Lowell, MA, USA), and 160 μL of erythrocytes was added. After incubation at 37 °C for 30 min, the samples were centrifuged, and the absorbance of the supernatant was measured at 450 nm using a multi-well microplate reader (SpectraMax M5; Molecular Devices, Sunnyvale, CA, USA) and compared with the 100% haemolysis caused by 0.1% Triton X-100. The percentage of haemolysis was calculated according to the equation:

$$Haemolysis\left(\%\right)=\frac{A_{sample}-A_{blank}}{A_{triton}-A_{blank}}\times 100$$

### 4.4. Cytotoxicity of the Peptides

The cytotoxicity of the peptides against BEAS-2B cells was evaluated using a standard CCK-8 assay. BEAS-2B cells were cultured in RPMI 1640 supplemented with 10% (*v*/*v*) FBS, 2 mM l-glutamine, 100 U/mL penicillin, and 100 mg/mL streptomycin, and maintained in a humidified incubator with 5% CO_2_ at 37 °C. Subsequently, BEAS-2B cells (5 × 10^3^ cells/well) were seeded in 96-well plates (Corning Inc., Lowell, MA, USA) and incubated overnight. The diluted peptides were introduced to BEAS-2B cells and incubated for 1 h. Subsequently, 10 μL of CCK-8 solution was added and incubated for 2 h. The absorbance at 450 nm was then quantified using a microplate reader (SpectraMax M5; Molecular Devices, Sunnyvale, CA, USA). Cell viabilities were determined using the provided equation:

$$Cell\;Viability\left(\%\right)=\frac{A_{sample}-A_{blank}}{A_{triton}-A_{blank}}\times 100$$

### 4.5. Antimicrobial Activity of the Peptides

The antibacterial activities of the peptides against three strains of Gram-negative bacteria and three strains of Gram-positive bacteria were evaluated using a modified version of the Clinical Laboratory and Standards Institute (CLSI) broth microdilution method, as previously described [19]. Briefly, the bacteria were cultured in liquid LB medium (comprising 10 g/L tryptone, 5 g/L yeast extract, and 10 g/L NaCl) at 37 °C until reaching mid-log phase. Subsequently, the bacterial concentration was adjusted to 1 × 10^6^ colony-forming units (CFU)/mL. The peptides were then diluted serially with phosphate-buffered saline (PBS), followed by the addition of 50 μL of the diluted peptides to 50 μL of the bacterial suspension in a 96-well plate (Corning Inc., Lowell, MA, USA). After incubating at 37 °C for 18 to 20 h, the absorbance of each well was measured using a multi-well microplate reader (SpectraMax M5; Molecular Devices, Sunnyvale, CA, USA) at 600 nm. The minimal inhibitory concentration (MIC) was defined as the lowest concentration at which the peptide completely suppressed bacterial growth.

### 4.6. Cell Membrane Damage Induced by the Peptides

Peptide-induced membrane damage was evaluated by quantifying the influx of propidium iodide (PI). BEAS-2B cells (1 × 10^6^ cells in 50 μL) were exposed to a final concentration of 6.25 μM peptide and incubated at 37 °C for 30 min, followed by PBS washing and re-suspension in PI solution (dissolved in PBS) at a final concentration of 2 μg/mL. Bacteria were cultured to mid-log phase at 37 °C, then diluted to an OD600 of 0.5 in phosphate-buffered saline (PBS). Antimicrobial peptides (AMPs) were introduced to 50 μL bacterial suspension at a final concentration of 6.25 μM for *E. coli* and *S. aureus*, followed by a 30-min incubation period at 37 °C. The bacteria were harvested and re-suspended in PI solution, with fluorescence signal analysis conducted using flow cytometry (Cytomics FC 500, Beckman Coulter, Indianapolis, IN, USA).

### 4.7. Inhibition of Nitric Oxide (NO) Production of SAAP-148 and Its Analogues

RAW264.7 cells were cultured in DMEM supplemented with 10% (*v*/*v*) FBS, 2 mM l-glutamine, 100 U/mL penicillin, and 100 mg/mL streptomycin, and maintained in a humidified incubator with 5% CO_2_ at 37 °C. Subsequently, RAW264.7 cells (1.5 × 10^4^ cells/well) were seeded in 96-well plates (Corning Inc., Lowell, MA, USA) and incubated overnight. LPS (100 ng/mL)-treated RAW264.7 cells were incubated with various concentrations of SAAP-148 and its analogues (6.25–25 μg/mL). After incubation for 24 h, the culture supernatant was collected, and NO assay was performed according the instruction. The absorbance of the sample was measured at 550 nm.

### 4.8. Statistical Analysis

Experimental data were encoded in Graphpad 6.0 and presented as mean ± SD. Statistical analyses were performed using unpaired *t*-tests.

## 5. Conclusions

In conclusion, a series of designed AMPs with an increased net positive charge were designed and evaluated for their efficacy against erythrocytes, eukaryotic cells, and bacteria. The substitution of lysine resulted in an increase in the overall charge of SAAP-148 and a decrease in its overall hydrophobicity and hyd. moment, except for SAAP-10, where an analogue substitution occurred at the 18th residue. The replacement of lysine led to a reduction in hemolytic activity and cytocity towards BEAS-2B cells compared to SAAP-148, with slightly higher haemolysis rates observed in SAAP-11 and SAAP-13. Additionally, the arrangement of positively charged residues in the peptide significantly influences its antimicrobial activity. Our findings suggest that the positioning of a positively charged residue has a significant impact on the biophysical properties and selectivity of the peptide. These discoveries provide valuable insights for the design and optimization of antimicrobial peptides, which will be advantageous for the future development of antimicrobial agents.

## Figures and Tables

**Table 1:** Sequence and molecular weight of SAAP-148 and its analogues.

Peptides	Sequence	MW by MS ^1^	Theroretical MW ^2^
LL-37	LLGDFFRKSKEKIGKEFKRIVQRIKDFLRNLVPRTES.NH_2_	4493.3	4493.32
SAAP-148	LKRVWKRVFKLLKRYWRQLKKPVR.NH_2_	3224.7	3225.03
SAAP-1	**K**KRVWKRVFKLLKRYWRQLKKPVR.NH_2_	3239.4	3240.04
SAAP-2	LKR**K**WKRVFKLLKRYWRQLKKPVR.NH_2_	3252.9	3254.06
SAAP-3	LKRV**K**KRVFKLLKRYWRQLKKPVR.NH_2_	3166.8	3166.98
SAAP-4	LKRVWKR**K**FKLLKRYWRQLKKPVR.NH_2_	3254	3254.06
SAAP-5	LKRVWKRV**K**KLLKRYWRQLKKPVR.NH_2_	3206	3206.2
SAAP-6	LKRVWKRVFK**K**LKRYWRQLKKPVR.NH_2_	3240	3240.04
SAAP-7	LKRVWKRVFKL**K**KRYWRQLKKPVR.NH_2_	3240	3240.04
SAAP-8	LKRVWKRVFKLLKR**K**WRQLKKPVR.NH_2_	3189.6	3190.2
SAAP-9	LKRVWKRVFKLLKRY**K**RQLKKPVR.NH_2_	3166.4	3166.98
SAAP-10	LKRVWKRVFKLLKRYWR**K**LKKPVR.NH_2_	3223.9	3225.07
SAAP-11	LKRVWKRVFKLLKRYWRQ**K**KKPVR.NH_2_	3240	3240.4
SAAP-12	LKRVWKRVFKLLKRYWRQLKK**K**VR.NH_2_	3255.7	3256.08
SAAP-13	LKRVWKRVFKLLKRYWRQLKKP**K**R.NH_2_	3253.8	3254.06

^1^ Molecular weight (MW) as measured by mass spectroscopy (MS), ^2^ The calculated molecular weight.

**Table 2:** Biophysical properties of SAAP-148 and its analogues.

Peptides	Hydrophobicity (H) ^1^	Hyd. Moment (μH) ^2^	Charge (z) ^3^	Rt (min) ^4^
LL-37	0.20081	0.52117	4	11.125
SAAP-148	0.30083	0.82272	11	12.416
SAAP-1	0.18875	0.71063	12	11.419
SAAP-2	0.20875	0.78127	12	12.532
SAAP-3	0.16583	0.72391	12	11.208
SAAP-4	0.20875	0.73708	12	9.96
SAAP-5	0.185	0.80996	12	10.681
SAAP-6	0.18875	0.81149	12	9.213
SAAP-7	0.18875	0.71815	12	12.897
SAAP-8	0.21958	0.76261	12	13.475
SAAP-9	0.16583	0.76344	12	11.941
SAAP-10	0.26875	0.82908	12	13.69
SAAP-11	0.18875	0.71063	12	15.926
SAAP-12	0.22958	0.7899	12	16.604
SAAP-13	0.20875	0.75411	12	16.867

^1^ Peptide hydrophobicity, ^2^ nean relative hydrophobic moment, and ^3^ peptide charge were determined at the following website: https://heliquest.ipmc.cnrs.fr/ (accessed on 12 March 2024). ^4^ Retention time measured by reverse-phase HPLC.

**Table 3:** Antimicrobial activities of SAAP-148 and its analogues against bacteria.

Peptides	MIC (μM) ^1^			GM to G^− 2^	MIC (μM) ^1^		GM to G^+ 2^
	*E. coli*	*P. aeruginosa*	*K. pneumoniae*		*S. aureus*	*S. epidermidis*	
LL-37	1.56	12.50	100.00	12.49	100.00	100.00	100
SAAP-148	6.25	3.13	6.25	4.96	50.00	3.13	12.51
SAAP-1	3.13	3.13	6.25	3.94	50.00	3.13	12.51
SAAP-2	3.13	3.13	12.50	4.97	50.00	3.13	12.51
SAAP-3	1.56	3.13	6.25	3.12	100.00	3.13	17.69
SAAP-4	1.56	3.13	100.00	7.87	100.00	3.13	17.69
SAAP-5	1.56	6.25	6.25	3.94	25.00	3.13	8.85
SAAP-6	1.56	1.56	6.25	2.48	25.00	1.56	6.24
SAAP-7	3.13	3.13	100.00	9.93	100.00	12.50	35.36
SAAP-8	1.56	1.56	25.00	3.93	25.00	1.56	6.24
SAAP-9	3.13	1.56	12.50	3.94	50.00	3.13	12.51
SAAP-10	6.25	6.25	25.00	9.92	50.00	3.13	12.51
SAAP-11	6.25	6.25	100.00	15.75	50.00	3.13	12.51
SAAP-12	12.50	12.50	100.00	25	25.00	6.25	12.5
SAAP-13	6.25	12.50	25.00	12.5	50.00	6.25	17.68

^1^ Minimum inhibitory concentration (MIC) was determined as the lowest concentration of peptide that prevented visible turbidity. ^2^ The geometric mean (GM) of the peptide minimum inhibitory concentrations (MICs) against Gram-negative and Gram-positive bacteria was calculated, separately.

**Table 4:** Antimicrobial and hemolytic activities of SAAP-148 and its analogues against bacteria and human erythrocytes.

Peptides	MIC (GM) ^1^	MHC ^2^	TI (MHC/MIC) ^3^	Fold ^4^
LL-37	28.72	100.00	3.48	
SAAP-148	7.18	0.78	0.11	
SAAP-1	6.25	0.78	0.12	1.1
SAAP-2	7.18	0.78	0.11	1
SAAP-3	6.25	50.00	8.00	72.73
SAAP-4	10.88	100.00	9.19	83.55
SAAP-5	5.44	6.25	1.15	10.45
SAAP-6	3.59	6.25	1.74	15.82
SAAP-7	16.49	100.00	6.06	55.09
SAAP-8	4.74	3.13	0.66	6
SAAP-9	6.25	6.25	1.00	9.09
SAAP-10	10.88	0.78	0.07	0.64
SAAP-11	14.36	0.78	0.05	0.45
SAAP-12	18.95	0.78	0.04	0.36
SAAP-13	14.36	0.78	0.05	0.45

^1^ The geometric mean (GM) of the peptide minimum inhibitory concentrations (MICs) against all five bacterial strains was calculated. ^2^ The minimum haemolytic concentration (MHC) is defined as the concentration that caused 10% haemolysis of erythrocytes. In cases where no detectable haemolytic activity was observed at 100 μM, a value of 200 μM was used to calculate the therapeutic index. ^3^ The therapeutic index (TI) is determined by the ratio of the MHC to the geometric mean of MIC (GM), with larger values indicating greater cell selectivity. ^4^ Fold represents the TI of analogue changes compared with SAAP-148.

**Table 5:** The membrane damage of BEAS-2B cells, *E. coli* and *S. aureus* treated by SAAP-148 and its analogues, as measured by the proportion of PI-positive cells.

Peptides	BEAS-2B	*E.* *coli*	*S. aureu* *s*
LL-37	31.2	78.9	46.9
SAAP-148	76.2	79.9	68
SAAP-1	62.1	75.8	60.5
SAAP-2	79.4	79.2	64.2
SAAP-3	36.4	54.1	48.5
SAAP-4	48.6	62	38
SAAP-5	54	73.2	52.4
SAAP-6	58.1	72	56.2
SAAP-7	30.1	34.9	21.1
SAAP-8	55.6	57.7	51.6
SAAP-9	81.7	53.4	48.8
SAAP-10	87.1	79.1	60.9
SAAP-11	53.3	69.2	65.5
SAAP-12	32.8	70.3	60.5
SAAP-13	31.7	75.6	64.5

## Data Availability

Data are contained within the article and Supplementary Materials.

## Supplementary Materials

The following supporting information can be downloaded at: https://www.mdpi.com/article/10.3390/ijms252111776/s1.
