# Supplementary material for: Designing Analogs of SAAP-148 with Enhanced Antimicrobial and Anti-LPS Activities
Source: Int J Mol Sci. 2024 Nov 1;25(21):11776. doi: 10.3390/ijms252111776 (PMC11546786; doi:10.3390/ijms252111776)
Supplement: Supplementary file 1 [file ijms-25-11776-s001.zip › ijms-3252197-supplementary.pdf]

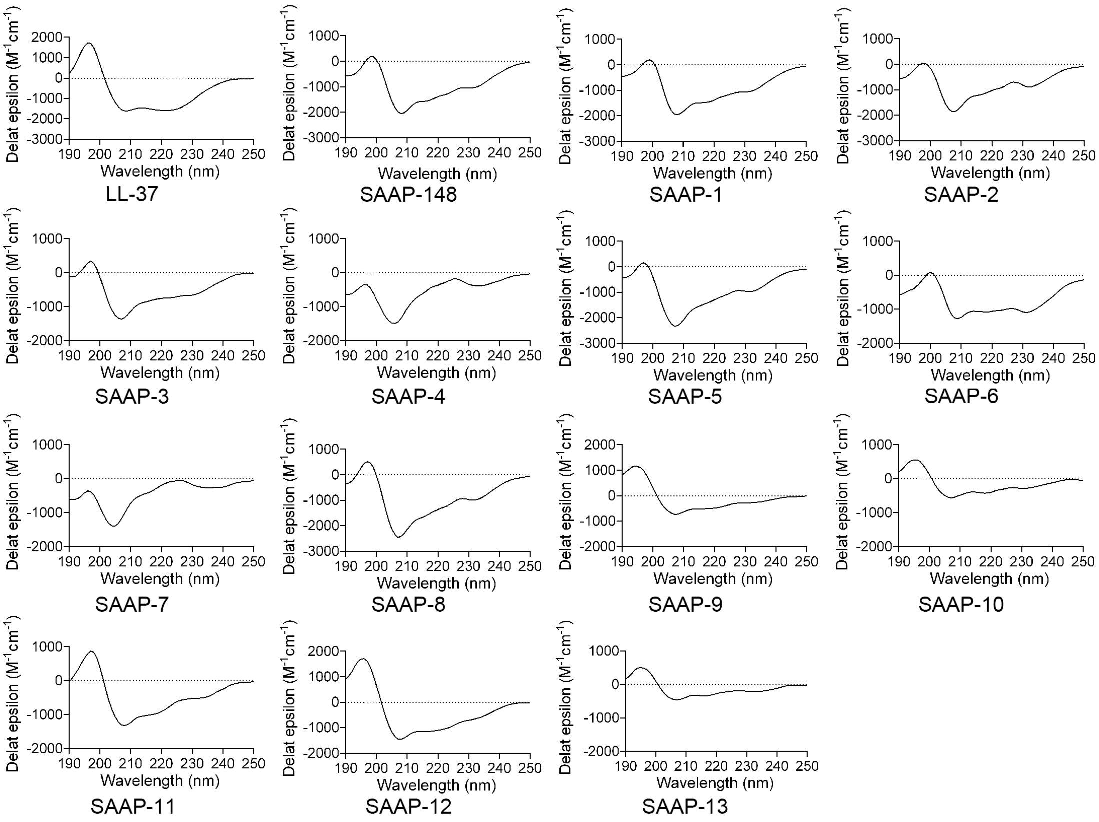

Figure S1. Circular dichroism of SAAP-148 and its analogues recorded on a JASCO J-1500 spectropolarimeter and analysized using BeStSel server.

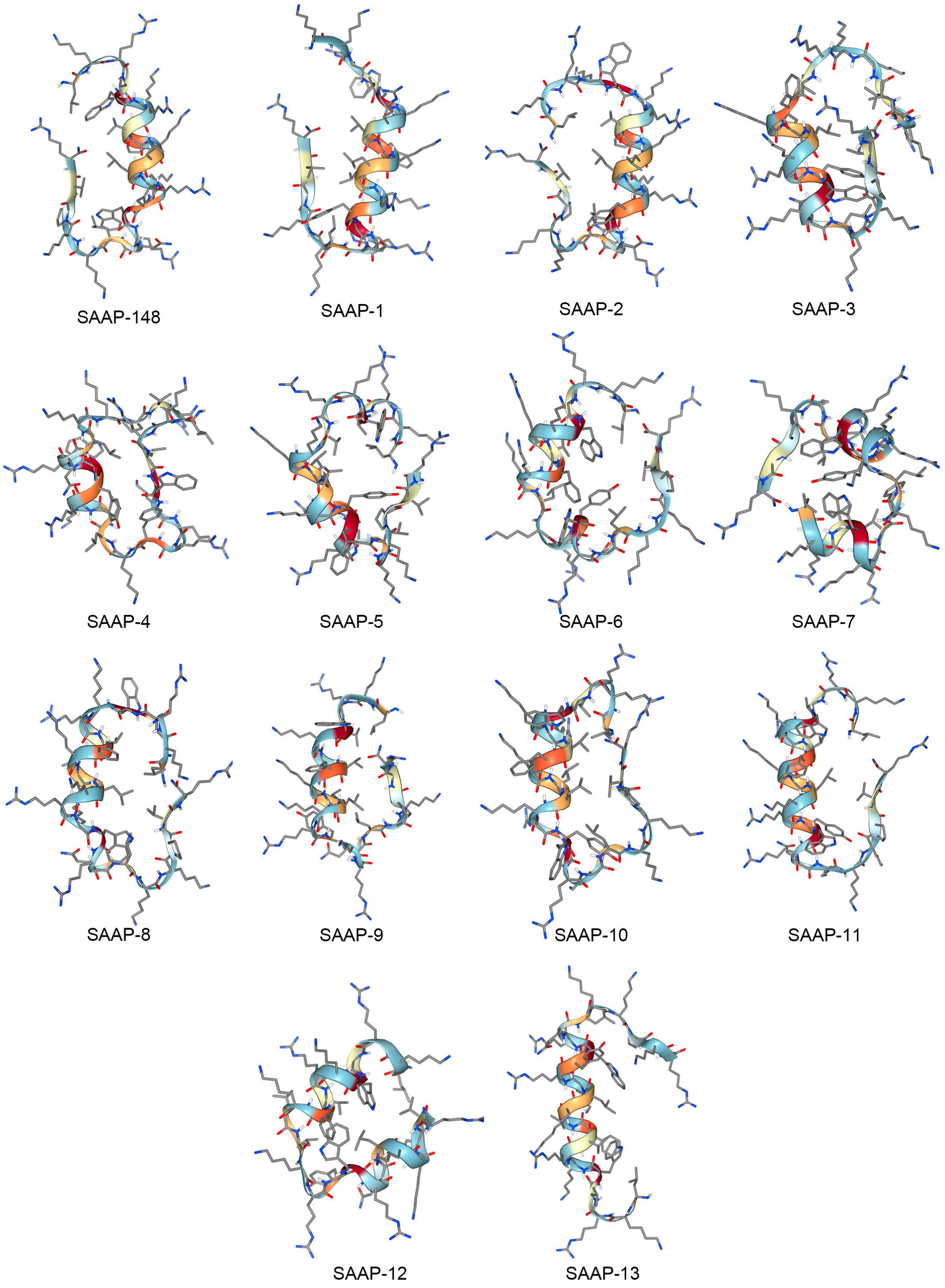

Figure S2. 3D structure of SAAP-148 and its analogues predicted using PEP-FOLD4 servers (https://bioserv.rpbs.univ-paris-diderot.fr/services/PEP-FOLD4/ Parameter settings: Generator:ts3, Number of models:200,Use Debye-Huckel contribution:Yes,Solvent pH:7,Ionic strenghth(mM):150)

Table S1. The percentage of secondary structure found for each peptide in 30 μM SDS. Secondary structure was calculated using BeStSel server.

Peptides	α-Helix	β-Antiparallel	β-Parallel	Turn	Others
LL-37	100	0	0	0	0
SAAP-148	93.5	6.5	0	0	0
SAAP-1	93.5	6.5	0	0	0
SAAP-2	93.5	6.5	0	0	0
SAAP-3	100	0	0	0	0
SAAP-4	50.9	45.5	0	0	3.6
SAAP-5	100	0	0	0	0
SAAP-6	84.4	15.6	0	0	0
SAAP-7	48.3	48.3	0	0	3.4
SAAP-8	100	0	0	0	0
SAAP-9	100	0	0	0	0
SAAP-10	100	0	0	0	0
SAAP-11	100	0	0	0	0
SAAP-12	100	0	0	0	0
SAAP-13	98.8	0	0	0	1.2

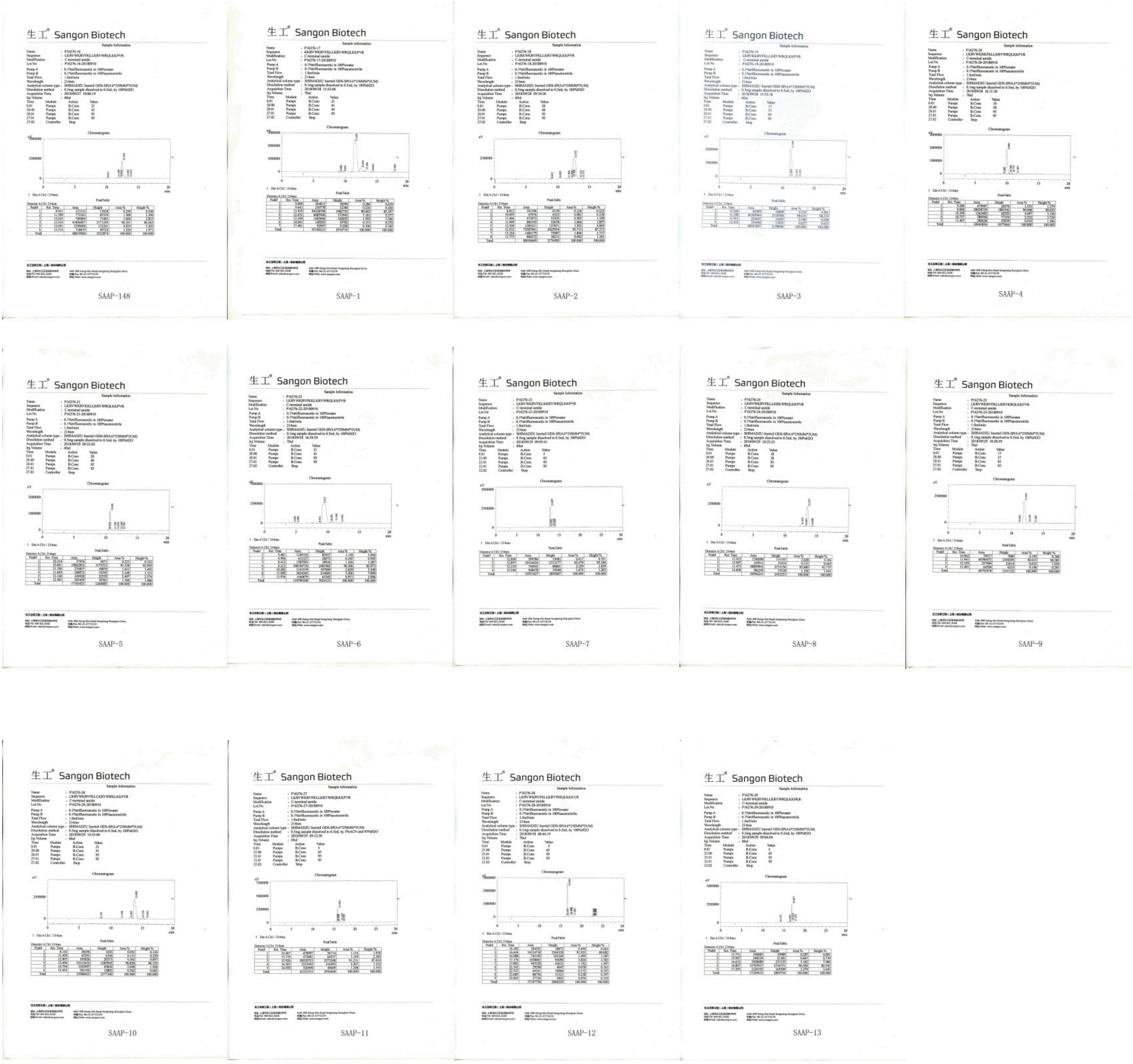

Figure S3. The HPLC of all AMPs

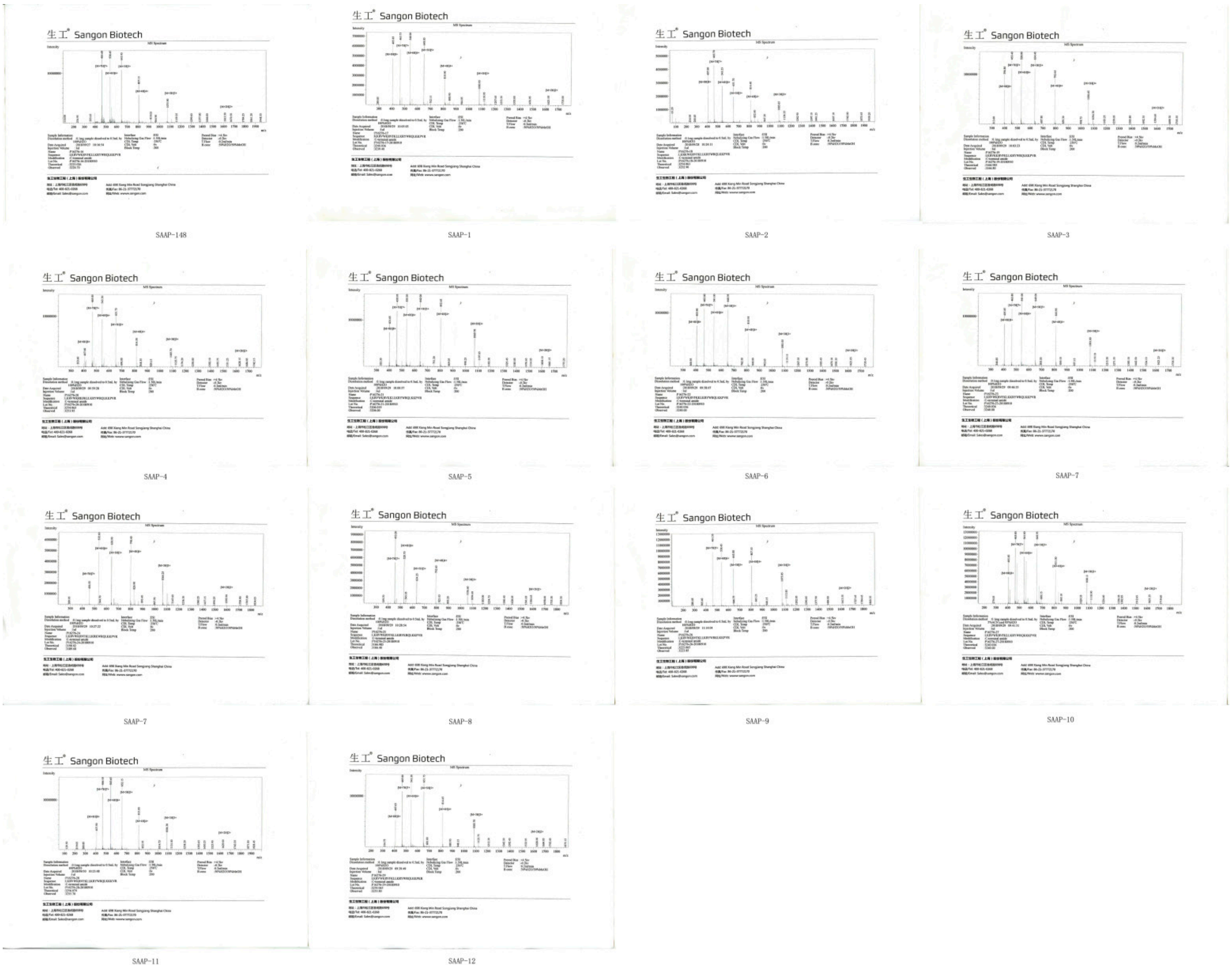

Figure S4. The ESI-MS of all AMPs
